# Knowledge, Attitude, and Practice of Adult Diabetics Regarding Diabetic Foot Ulcers: A Cross-Sectional Study in Saudi Arabia

**DOI:** 10.7759/cureus.53356

**Published:** 2024-01-31

**Authors:** Safa H Alkalash, Faisal H Alnashri, Amnah I Alnashri, Khadijah A Alghubayshi, Manar A Alsumaydi, Wael S Alzubaidi, Sarah M Alshuqayfi, Fuad M Alkudaysi, Naeem F Qusty

**Affiliations:** 1 Community Medicine and Health Care, Umm Al-Qura University, Al-Qunfudah, SAU; 2 Family Medicine, Menoufia University, Shibin El Kom, EGY; 3 College of Medicine, Umm Al-Qura University, Al-Qunfudah, SAU; 4 Pediatric Department, South Al-Qunfudah Hospital, Al-Qunfudah, SAU; 5 Laboratory Medicine Department, Faculty of Applied Medical Sciences, Umm Al-Qura University, Makkah, SAU

**Keywords:** saudi arabia, al-qunfudah, practice, knowledge, diabetes, attitude

## Abstract

Background

Diabetic foot ulcers (DFUs) are one of the most common and fatal complications of diabetic patients with uncontrolled diabetes mellitus (DM) that may end with their feet amputation. These complications can be prevented through the adherence of diabetic patients to their diabetes management plan and by educating them about risk factors, complications of diabetic foot, and proper foot care. To develop effective health education programs to educate diabetic patients and caregivers regarding diabetic foot and its effective care, we should first identify gaps in patients' knowledge and perception of diabetic foot and evaluate their practice of foot care.

Objectives

This study aimed to evaluate knowledge and attitude toward DFUs and the practice of foot care among adult diabetic patients attending Al-Qunfudah Diabetes Center, Saudi Arabia, from October 2022 to March 2023.

Methods

A total of 403 adult diabetic patients were recruited in this cross-sectional study during their attendance at the diabetes center in Al-Qunfudah district, Saudi Arabia. The study researchers conducted a physical face-to-face interview with each diabetic patient using a validated questionnaire with closed-ended questions to collect their responses regarding their knowledge and attitude toward DFUs and their behavior toward foot care. The collected data were analyzed using IBM SPSS Statistics for Windows, version 23 (released 2015; IBM Corp., Armonk, New York, United States).

Results

Out of 403 diabetics, 50.4% (n = 203) had inadequate knowledge (knowledge score < 80%), 46.4% (n = 187) had negative attitudes (attitude score < 80%) toward DFUs, and the majority could not practice foot care well, with 77.7% (n = 313) giving a practice score less than 80%. Seventy patients (17.4%) self-reported a history of DFUs. Predictors of good knowledge about DFUs among diabetics include age between 30 and 59 years (odds ratio (OR) = 2.942, confidence interval (CI) 95% = 1.695-2.107, p < 0.001), marriage (OR = 3.101, CI 95% = 1.893-5.079, p < 0.001), working (OR = 5.325, CI 95% = 3.019-9.389, p < 0.001), diploma education (OR = 8.205, CI 95% = 3.332-20.203, p < 0.001), managing DM with oral drugs (OR = 2.219, CI 95% = 1.399-3.519, p < 0.001), and having no DFUs (OR = 2.712, CI 95% = 1.557-4.723, p < 0.001). Males were more likely to practice foot care well (OR = 1.925, CI 95% = 1.142-3.245, p = 0.013). Primary education (OR = 3.421, CI 95% = 1.655-7.073, p < 0.001) predicted appropriate foot care. Patients with DM for one to five years (OR = 1.995, CI 95% = 1.139-3.493, p = 0.016) and those on diet and metformin (OR = 2.133, CI 95% = 1.134-4.011, p = 0.019) were expected to have better foot care than other diabetics.

Conclusion

Diabetic patients in the Al-Qunfudah district of Saudi Arabia had inadequate knowledge and negative attitudes toward DFUs, and their foot care behaviors were inadequate. Furthermore, DFUs were self-reported in around 17% of diabetic patients. Specialized training programs are recommended to enhance knowledge regarding DFUs among diabetics and motivate and train them and caregivers on how to conduct proper foot care. These educational programs should target all diabetics, with an emphasis on those with DFUs, females, non-working patients, individuals who have had DM for a longer time, and illiterate diabetics. To understand the factors behind patients' negative attitudes toward diabetic foot, future qualitative research is required.

## Introduction

Diabetes mellitus (DM) is a metabolic disorder characterized by hyperglycemia and changes in carbohydrate, protein, and lipid metabolism [1]. Globally, there are 537 million diabetics, including 73 million in the Middle East and Northern Africa (MENA) region; by 2045, that number is expected to increase to 135.7 million [2]. The International Diabetes Federation MENA region, which comprises 21 nations and territories, including Saudi Arabia, reported that Saudi Arabia is one of the countries in the MENA region with the highest prevalence of DM, representing 17.7% of the adult Saudi population [2].

DM is the most common cause of disabilities and deaths globally, with complications of vascular diseases accounting for 26.8% of all cases [3]. Among these complications are diabetic foot and diabetic foot ulcers (DFUs), which are ulcerations that typically appear in the plantar portion of the foot [4]. A DFU is a serious indication of uncontrolled and persistent DM [4]. Although peripheral neuropathy, peripheral artery disease, and foot abnormalities are the main clinical risk factors for DFUs, there are other significant moderators of risk for DFUs and lower-extremity amputation, including geography, race, ethnicity, and socioeconomic status [5]. The lifetime risk of DFUs is 19% to 34%, and this percentage is rising as patients with DM live longer and have more medical complications. Health problems occur frequently following initial ulceration, with rates of recurrence of about 65% within three to five years, an average of 20% lifetime lower-extremity amputation rate, and a mortality rate after five years of 50-70% [6]. The prevalence of DFUs in Saudi Arabia ranged from 2.05% to 35.1% [7-9].

Patient education and annual diabetic foot assessments for peripheral vascular disease and neuropathy in healthcare facilities are preventive methods that can help prevent developing DFUs or control the severity of these ulcers [10]. In addition, management that includes strict glycemic control along with proper foot care can improve the patient's quality of life and prevent further morbidity that could lead to amputation or subsequent death [11]. Some studies suggest that patient education could positively influence patient behavior toward foot care in the short term [12-13]. Furthermore, inadequate knowledge about foot care is associated with poor foot care [14-15].

The knowledge, attitude, and practice regarding diabetic foot among diabetic patients have been assessed through several studies conducted around different regions of Saudi Arabia [16-22]. Al-Hariri and his colleagues in Dammam reported good knowledge and favorable attitudes toward diabetic foot, while a high percentage of the participants ignored critical information and instructions before buying their shoes [16]. Shamim and his colleagues in Alkharj discovered that diabetics have a good understanding of diabetes and related foot issues and a favorable attitude toward its management. However, they were lagging in the practices necessary for diabetic foot management [17]. Despite the importance of recognizing the extent of diabetic patients awareness regarding DFUs and how they perceive and their practice of foot care, no previous studies evaluated these critical issues among diabetic patients in the Al-Qunfudah district of Saudi Arabia; therefore, this study was applied.

## Materials and methods

Study design

This study employed a cross-sectional research design to assess diabetic patients' knowledge and attitudes toward DFUs and their practices of foot care, addressing them using a validated questionnaire administered via face-to-face interview.

Study setting

The research was carried out at Al-Qunfudah Diabetes Center, a specialized healthcare facility affiliated with Al-Qunfudah General Hospital, in the Al-Qunfudah district of Saudi Arabia. The center provides different healthcare services related to DM, including examinations, investigations, counseling, management, and follow-up for all diabetic patients. This center offers DM patients outpatient healthcare services, but inpatient hospitalization is not available. Patients who need hospitalization can be admitted to Al-Qunfudah General Hospital.

Sample size calculation

The Epi Info tool (Centers for Disease Control and Prevention (CDC), Atlanta, Georgia, USA) was used to estimate the minimum required sample size. The estimation was based on the total population of the Al-Qunfudah district (300,516) and a Saudi survey [14] that found that 55.1% of diabetic patients had good awareness about diabetic foot, with a 5% margin of error and a 95% confidence interval (CI). The minimum acceptable sample size was determined to be 384.

Study sample criteria

This study targeted male or female diabetic patients at the age of 18 or more, whether Saudi or non-Saudi, with any type of DM, provided that they were competent and able to give informed consent to participate in the study. Meanwhile, the exclusion criteria involved diabetic patients who refused to participate and those with cognitive impairments that hindered their ability to give informed consent or answer the questionnaire.

Data collection

Data were collected using a validated questionnaire, which was developed based on existing literature [7,8,13,14]. The questionnaire was administered in Arabic, the local language of the participants. It was subdivided into five domains: (i) The first domain consisted of items to assess the sociodemographic profile, such as age, gender, nationality, education, occupation, and marital status. (ii) The second section involved questions about their history of smoking; any comorbidity with other chronic illnesses; family history and duration of DM; current medications for managing DM; foot sensation; whether they had any abnormal sensations, such as tingling, numbness, or absence of normal sensation; and whether they were having DFUs (recent or in the past) or not. (iii) The third part consisted of items to assess their knowledge regarding diabetic foot, covering various aspects, such as foot ulcers, blood circulation, sensory impairment, and the importance of foot examination. (iv) The fourth section inquired about patients’ attitudes toward diabetic foot and their understanding of their willingness to adopt preventive measures and seek medical advice when needed. (v) The fifth and last portion of the survey assessed the patients' self-reported practices regarding their foot care, which were recorded through questions related to daily foot examination, washing, footwear choices, and nail care.

The survey was digitalized using the Google Form application (Google LLC, Mountain View, California, United States) to allow easy data collection. Then, it was pretested in a pilot study in order to determine whether its contents would be understandable by the study participants and to assess its validity and reliability. During the pilot study, the researchers conducted interviews with diabetic patients and shared the survey link on WhatsApp (Meta Platforms, Inc., Menlo Park, California, United States) during their attendance at Al-Qunfudah Diabetes Center. This pilot involved a total of 38 patients (representing 10% of the estimated sample size). The validity of the survey was assessed to ensure its clarity, comprehensiveness, and applicability through expert input from healthcare professionals specializing in diabetes care (family medicine, internal medicine, and general surgery). The reliability of the survey was then tested through an evaluation of the responses of the pilot participants. Cronbach's alpha was used for determining the survey's reliability; it is frequently expressed as a number ranging from 0.00 to 1.0. A score of 0.00 indicates no measurement consistency, whereas 1.0 represents full measurement consistency. The range that is acceptable is 0.70 to 0.90, or greater, depending on the type of research. Cronbach's alpha of 0.70 is suitable for exploratory research, while 0.80 and 0.90 are appropriate for basic research and applied scenarios [23]. In this study, the Cronbach's alpha values were 0.86 for stated knowledge, 0.81 for attitude, and 0.88 for foot care practice, which indicated that the survey is reliable. The findings obtained from the pilot study were not included in the main study results.

The necessary research data were collected by interviewing diabetic patients during their attendance at Al-Qunfudah Diabetes Center. Over the course of six months, from October 2022 to March 2023, the researchers performed interviews with the patients after explaining the study and taking their informed consent to be part of the study. During this interview, they were asked whether they had ever been diagnosed with DFUs, and researchers inspected their feet for any ulcers or skin lesions. They then sent the survey link to their WhatsApp in order to submit their answers about their knowledge, attitudes, and practice of diabetic foot. The data collectors assisted illiterate patients and those who were unable to submit their replies.

After completing the data collection phase, the data were evaluated and refined to identify incomplete responses. A total of 416 questionnaires were collected. Thirteen incomplete surveys were discarded, and 403 surveys were valid and analyzed to obtain the final study results. The response rate was 97.4%; a high response rate means that the sample is more likely to be representative of the target population.

Data analysis

Data analysis was done by IBM SPSS Statistics for Windows, version 23 (released 2015; IBM Corp., Armonk, New York, United States). Descriptive statistics are expressed in frequencies and percentages for categorical data. The total knowledge score has been calculated by summing all its items. It was composed of 13 questions; the correct answer was scored "1," while the incorrect and do not know were given a "0." By using 80% as a cutoff point, the level of patients' knowledge regarding DFUs was evaluated; patients were considered to have good knowledge if the score was at 80% or higher [24], while those below 80% were categorized as having inadequate knowledge, and the same was applied for attitude and practice scores. Differences in knowledge, attitudes, and practices based on socioeconomic characteristics were tested using the chi-squared test for normally distributed variables. Non-parametric data were evaluated using Fisher's exact test. A binary regression model was used to identify independent significant predictors of knowledge, attitudes, and practices in the study sample, along with their odds ratios (ORs) and 95% confidence intervals (CIs). Values with a p-value less than 0.05 are regarded as significant.

Ethical considerations

Ethical approval for the study was obtained from the research ethics committee of Umm Al-Qura University (approval number: HAPO-02-K-012-2022-09-1176). In order to obtain informed consent, each participant was informed about the study objectives and procedure for data collection, ensuring that all personal identification data were kept confidential. To maintain privacy, each participant was interviewed, and their feet were inspected individually. All information was carefully handled to ensure its confidentiality.

## Results

A total of 403 diabetic patients were recruited for this study. In terms of age, about half of the participants were between 30 and 59 years old, constituting 55.6% (n = 224) of the total sample. Regarding sex, the study had a higher representation of female participants, accounting for 77.4% (n = 312) of the total sample. Nationality-wise, the vast majority of participants were Saudi citizens, making up 97.5% (n = 393) of the sample. The majority of the participants were married, constituting 65.3% (n = 263) of the sample. The largest proportion were non-working individuals, comprising 65.3% (n = 263) of the sample. Concerning educational level, the highest proportion (23.6%, n = 95) were illiterate, which may negatively affect the knowledge level among the studied group, while individuals with primary and bachelor's degrees each represented 22.6% (n = 91) of the sample (Table 1).

**Table 1:** Sociodemographic characteristics of the study participants (n = 403)

Parameter		Frequency (%)
Age in years	18-29	76 (18.9%)
	30-59	224 (55.6%)
	60 and above	103 (25.6%)
Sex	Female	312 (77.4%)
	Male	91 (22.6%)
Nationality	Saudi	393 (97.5%)
	Non-Saudi	10 (2.5%)
Marital status	Widowed	41 (10.2%)
	Single	99 (24.6%)
	Married	263 (65.3%)
Occupational status	Student	46 (11.4%)
	Non-working	263 (65.3%)
	Working	94 (23.3%)
Educational level	Illiterate	95 (23.6%)
	Primary education	91 (22.6%)
	Bachelor’s degree	91 (22.6%)
	Secondary education	53 (13.2%)
	Diploma	47 (11.7%)
	Master’s degree	4 (1.0%)
	Intermediate education	22 (5.5%)

Based on the medical histories of the research participants, it was found that 89.8% (n = 362) of them were non-smokers. Hypertension (HTN) was the most prevalent comorbid chronic illness, affecting 36.7% (n = 148) of the individuals. The positive family history of DM was high, representing 76.4% (n = 308). A comparatively uniform distribution of diabetes duration was noted in the subjects: 33.5% (n = 135) of those with diabetes for one to five years, 32.5% (n = 131) for six to 10 years, and 34% (n = 137) for 11 years or longer. In terms of diabetes therapy, insulin injection was the most commonly used treatment modality among 222 (55.1%) participants, whereas 29% (n = 117) used oral hypoglycemic pills (sulfonylurea, thiazolidinediones, and others), and 15.9% (n = 64) followed a healthy diet and oral metformin. Foot ulcers were self-reported by 17.4% (n = 70) of the participants. Concerning foot sensation, 75.7% of the participants (n = 305) reported having normal foot sensation without any tingling, burning, soreness, or shooting pains (Table 2).

**Table 2:** Medical history and complaints of the study participants (n = 403) DM: diabetes mellitus, HTN: hypertension

Parameter		Frequency (%)
Smoking status	Former smoker	20 (5.0%)
	Non-smoker	362 (89.8%)
	Current smoker	21 (5.2%)
Chronic diseases	Asthma	14 (3.5%)
	HTN	148 (36.7%)
	Hypothyroidism	13 (3.2%)
	Ischemic heart disease	31 (7.7%)
	Dyslipidemia	30 (7.5%)
	None	167 (41.4%)
Family history of DM	No	91 (22.6%)
	Don't know	4 (1.0%)
	Yes	308 (76.4%)
Duration of DM in years	One to five	135 (33.5%)
	Six to 10	131 (32.5%)
	More than 10	137 (34.0%)
DM management	Insulin	222 (55.1%)
	Diet control and metformin	64 (15.9%)
	Oral hypoglycemic medications	117 (29.0%)
Foot ulcers	No	333 (82.6%)
	Yes	70 (17.4%)
Foot sensation	No	81 (20.1%)
	Unsure	17 (4.2%)
	Yes	305 (75.7%)

Regarding the knowledge items, a noteworthy percentage of participants (74.7%, n = 301) stated that individuals with DM have a higher risk of developing foot ulcers. A comparable pattern was noted regarding the sensory impairment of diabetic patients' feet, as identified by 82.9% (n = 334) of participants. The vast majority of participants (93.1%, n = 375) accurately identified that taking medicine on a regular basis lowers the risks associated with diabetes, and 91.8% (n = 370) of them indicated that wounds and ulcers in the diabetic patients' feet may not heal quickly. On the other hand, certain regions lacked sufficient expertise. For example, 67.5% (n = 272) of the participants recognized that decreasing blood supply relates to diabetic foot problems, while only 57.6% (n = 232) of individuals correctly identified the link between smoking and decreased blood circulation in the feet. The mean score for the entire knowledge test came out to be 75.3± 21.9. The average proportion of accurate answers for all knowledge items is shown by this score. After assessing the participants' knowledge, it was found that 49.6% (n = 200) of them had good knowledge about diabetic foot. However, 50.4% (n = 203) of the participants had inadequate knowledge (Table 3).

**Table 3:** Knowledge of diabetic patients about diabetic foot (n = 403)

Knowledge items	Yes	No	Don't know
	n (%)	n (%)	n (%)
Diabetic patients are more likely to develop foot ulcers.	301 (74.7%)	46 (11.4%)	56 (13.9%)
Diabetic patients have reduced blood flow to their feet.	272 (67.5%)	26 (6.5%)	105 (26.1%)
Diabetic patients are more likely to develop sensory impairments in their feet.	334 (82.9%)	25 (6.2%)	44 (10.9%)
The feet of diabetic patients are more susceptible to becoming deformed.	318 (78.9%)	15 (3.7%)	70 (17.4%)
Gangrene of the foot is one of the complications of diabetes.	321 (79.7%)	15 (3.7%)	67 (16.6%)
Uncontrolled blood sugar levels may lead to foot deformities and disabilities.	269 (66.7%)	48 (11.9%)	86 (21.3%)
Taking medications regularly reduces diabetes complications.	375 (93.1%)	17 (4.2%)	11 (2.7%)
Wounds and ulcers in the diabetic patients' feet may not heal quickly.	370 (91.8%)	10 (2.5%)	23 (5.7%)
Diabetic foot complications increase if the diabetic patient also has hypertension.	348 (86.4%)	11 (2.7%)	44 (10.9%)
Smoking can decrease blood circulation in the feet, which increases the development of diabetic foot ulcers.	232 (57.6%)	30 (7.4%)	141 (35.0%)
Exercise improves blood circulation in the feet and protect from diabetic foot ulcers.	346 (85.9%)	0 (0.0%)	57 (14.1%)
Arterial stiffness contributes to diabetic foot ulcers.	233 (57.8%)	18 (4.5%)	152 (37.7%)
Infection contributes to diabetic foot ulcers.	235 (58.3%)	46 (11.4%)	122 (30.3%)

A considerable percentage of participants (72.5%, n = 292) expressed agreement that exercising regularly and making dietary changes can prevent further diabetes complications. Similarly, 69.5% (n = 280) of the participants accepted taking responsibility for daily foot examinations and seeking regular consultations with a foot care specialist. A majority of the participants agreed to utilize special shoes and indoor shoes (67.7%, n = 273, and 55.6%, n = 224, respectively) recommended by a foot care specialist to promote foot health. An overwhelming majority (87.8%, n = 354) had a proactive mindset toward managing diabetes effectively. The participants' responses to hypothetical situations were also assessed. For instance, in the case of developing plantar warts, the majority (85.6%, n = 345) opted to go to the hospital for appropriate medical attention. Similarly, if the participants experienced redness or bleeding between their toes, 79.2% (n = 319) decided to visit the hospital for evaluation and treatment. The overall attitude scores were calculated, yielding a mean score of 73.9 ± 24.5. At all, 53.6% (n = 216) of the participants demonstrated a positive attitude, while 46.4% (n = 187) exhibited a negative attitude toward diabetic foot (Table 4).

**Table 4:** Attitudes of diabetic patients toward diabetic foot (n = 403)

Attitude items	Agree	Neutral	Disagree
	n (%)	n (%)	n (%)
Exercising regularly and healthy eating habits may prevent further diabetes complications.	292 (72.5%)	22 (5.5%)	89 (22.1%)
A daily foot examination and regular consultation with a foot care specialist are beneficial procedures.	280 (69.5%)	35 (8.7%)	88 (21.8%)
It is important to use special shoes after consulting a foot care specialist.	273 (67.7%)	16 (4.0%)	114 (28.3%)
It is important to wear indoor shoes recommended by a foot care specialist.	224 (55.6%)	10 (2.5%)	169 (41.9%)
Adopt a healthy lifestyle with appropriate measures for diabetes.	354 (87.8%)	22 (5.5%)	27 (6.7%)
If you are developing plantar warts, you should consult a foot care specialist.	345 (85.6%)	26 (6.5%)	32 (7.9%)
If you experience redness or bleeding between your toes, you should consult a foot care specialist.	319 (79.2%)	9 (2.2%)	75 (18.6%)

About half of the participants (49.1%, n = 198) reported regularly examining their feet. A majority (62.3%, n = 251) of the participants checked the inside part of their shoes before wearing them and inspected their feet for signs resulting from shoes or socks (63.8%, n = 257). Regarding foot hygiene practices, an overwhelming majority (95.8%, n = 386) reported regularly washing their feet, and 87.6% (n = 353) trimmed their toenails regularly and straight across. However, it was observed that 18.9% (n = 76) of the participants checked the temperature of the water before washing their feet. Regarding the commitment of diabetics to wear comfortable and closed shoes, only 60.8% (n = 245) of the participants reported adherence to applying moisture to their feet. In addition, 40% (n = 161) of the participants reported wearing cotton socks regularly, indicating room for improvement in this aspect of foot care. The overall practice scores were calculated, resulting in a mean score of 53.9 ± 25.5. This score represents the average percentage of adherence to recommended diabetic foot care practices across all practice-related items. Finally, 22.3% (n = 90) of the participants demonstrated good foot care practice, while 77.7% (n = 313) exhibited poor practice (Table 5).

**Table 5:** Practice of foot care among diabetic patients (n = 403)

Practice items	No	Sometimes	Yes
	n (%)	n (%)	n (%)
Examine your feet daily.	195 (48.4%)	10 (2.5%)	198 (49.1%)
Check the inside of the shoes before wearing them.	142 (35.2%)	10 (2.5%)	251 (62.3%)
Inspect feet for any signs resulting from shoes or socks.	135 (33.5%)	11 (2.7%)	257 (63.8%)
Measure the size of your feet.	240 (59.6%)	15 (3.7%)	148 (36.7%)
Regularly wash your feet.	17 (4.2%)	0 (0.0%)	386 (95.8%)
Check the temperature of the water before washing your feet.	319 (79.2%)	8 (2.0%)	76 (18.9%)
Carefully dry the spaces between toes after washing.	158 (39.2%)	11 (2.7%)	234 (58.1%)
Apply moisturizer daily to the feet.	151 (37.5%)	7 (1.7%)	245 (60.8%)
Trim toenails regularly and straight across, not too short.	46 (11.4%)	4 (1.0%)	353 (87.6%)
Frequently walk barefoot outside.	334 (82.9%)	4 (1.0%)	65 (16.1%)
Wear comfortable, closed, and soft shoes.	146 (36.2%)	12 (3.0%)	245 (60.8%)
Wear cotton socks regularly.	238 (59.1%)	4 (1.0%)	161 (40.0%)
Replace shoes regularly, even without damage.	119 (29.5%)	4 (1.0%)	280 (69.5%)
Change responses daily.	143 (35.5%)	26 (6.5%)	234 (58.1%)
Receive advice before purchasing shoes.	244 (60.5%)	3 (0.7%)	156 (38.7%)
Visit the doctor regularly for foot checkups.	209 (51.9%)	9 (2.2%)	185 (45.9%)

**Figure 1:**
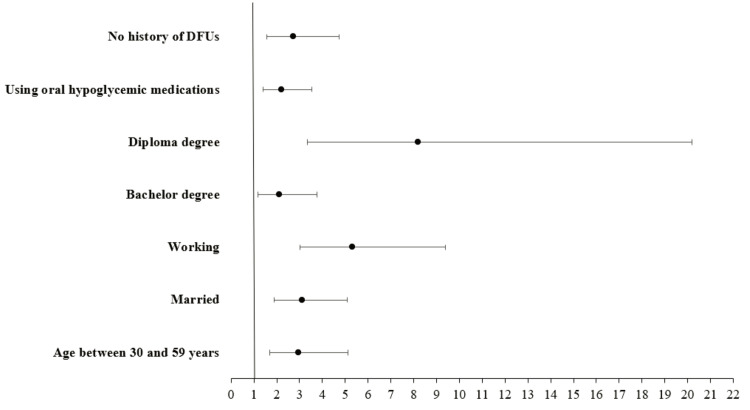
Forest plots for the odds ratios of predictors of good knowledge regarding diabetic foot ulcers among diabetic patients DFUs: diabetic foot ulcers

The following were predictors for good knowledge about DFUs among diabetic patients: having age between 30 and 59 years (OR = 2.942, CI 95% = 1.695-2.107, p < 0.001), being married (OR = 3.101, CI 95% = 1.893-5.079, p < 0.001), working individuals (OR = 5.325, CI 95% = 3.019-9.389, p < 0.001), holding a bachelor's degree (OR = 2.096, CI 95% = 1.168-3.761, p = 0.013) or diploma education (OR = 8.205, CI 95% = 3.332-20.203, p < 0.001), taking oral hypoglycemic medications (OR = 2.219, CI 95% = 1.399-3.519, p < 0.001), and having no DFUs (OR = 2.712, CI 95% = 1.557-4.723, p < 0.001) (Figure 1).

**Figure 2:**
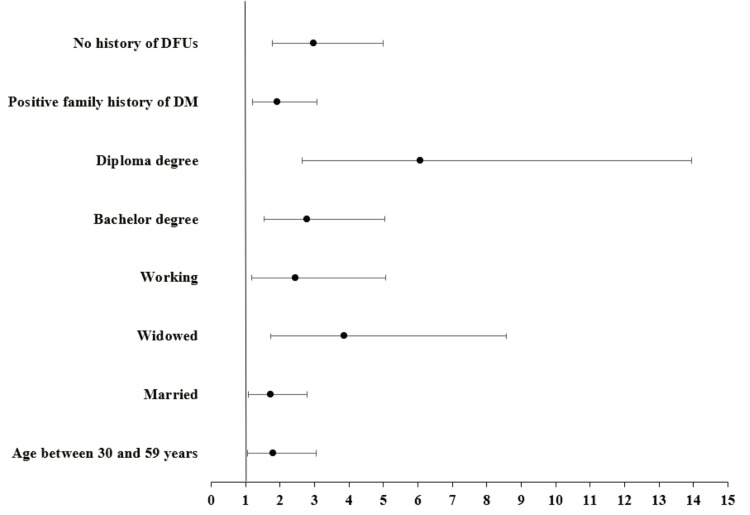
Forest plots for the odds ratios of the predictors of positive participants' attitudes toward diabetic foot ulcers. DM: diabetes mellitus; DFUs: diabetic foot ulcers

Predictors for a positive attitude toward diabetic foot were as follows: having an age between 30 and 59 years (OR = 1.802, CI 95% = 1.066-3.048, p = 0.028), being married (OR = 1.738, CI 95% = 1.089-2.776, p = 0.021), being widowed (OR = 3.858, CI 95% = 1.737-8.571, p < 0.001), working (OR = 2.445, CI 95% = 1.184-5.051, p = 0.016), holding a bachelor's degree (OR = 2.779, CI 95% = 1.532-5.043, p < 0.001) and diploma education (OR = 6.063, CI 95% = 2.634-13.955, p < 0.001), having a positive family history of DM (OR = 1.909, CI 95% = 1.189-3.068, p = 0.008), and free of DFUs (OR = 2.967, CI 95% = 1.768-4.979, p < 0.001) (Figure 2).

**Figure 3:**
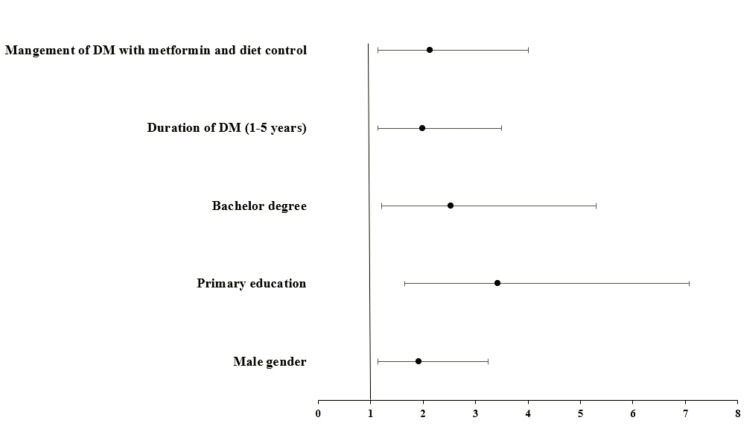
Forest plots for the odds ratios of the predictors of the best participants' practice of foot care. DM: diabetes mellitus; DFUs: diabetic foot ulcers

The data analysis detected that the best practice of foot care was predicted in male patients with an OR of 1.925, a CI 95% of 1.142-3.245, and a p-value of 0.013. Furthermore, having primary education (OR = 3.421, CI 95% = 1.655-7.073, p < 0.001) or having a bachelor's degree (OR = 2.523, CI 95% = 1.203-5.294, p = 0.014) were observed as predictors of good foot care. Patients with DM for one to five years (OR = 1.995, CI 95% = 1.139-3.493, p = 0.016) and those treated with diet and metformin (OR = 2.133, CI 95% = 1.134-4.011, p = 0.019) were predicted to possess better foot care than other diabetic patients (Figure 3).

## Discussion

DM is a common chronic disease with serious complications, one of which is diabetic foot [25]. DFU complications pose a significant burden on healthcare systems and can lead to amputations, decreased quality of life, and increased mortality rates [26]. Understanding the knowledge and attitude toward DFUs and foot care practices among diabetic patients is essential for implementing effective preventive measures and improving patient outcomes [27]. The current study aimed to assess the knowledge and attitude toward DFUs and the practice of foot care among adult diabetic patients attending Al-Qunfudah Diabetes Center in Saudi Arabia.

According to this study, 70 patients self-reported histories of DFUs represented 17.4%, which is higher than the global prevalence of 6.3% [28]. However, this prevalence rate of DFUs falls within the range of recorded rates in Saudi Arabia, ranging from 2.05% to 35.1% [7-9]. This diversity could be driven by disparities in healthcare access, DM treatment, and patient awareness of foot care practices in different regions, in addition to differences in study samples and methodologies. The higher prevalence rate of DFUs in Saudi Arabia than the global prevalence rate needs more investigations to identify accurate causes of this higher rate of DFUs, and more intensive care should be provided to diabetic patients to limit this critical issue.

As per this study's results, around 50% (n = 203) of the patients with DM showed poor knowledge of DFUs, which is in agreement with previous Saudi studies that declared a lower level of diabetic patients awareness regarding diabetic foot [19-22,27,29]. This result emphasizes the necessity of providing diabetic patients and their caregivers with targeted educational programs to increase their understanding of the mechanisms of diabetic foot and strategies to avoid its complications.

In this study, elderly diabetic patients and those with lower educational levels had poor knowledge about DFUs. This finding agrees with a prior Saudi study [29]. The gender of the patients had no significant relationship with their knowledge levels. This suggests that efforts to increase awareness should target all diabetic patients, regardless of gender. Furthermore, healthcare practitioners should design educational programs to meet the unique requirements of older people and those with lower levels of education [30].

The study assessed the attitude of diabetic patients toward DFUs and found that 53.6% (n = 216) of the participants had a positive attitude. This finding is much lower than what was recorded by studies in Dammam [16], Al-Kharj [17], and Al Madinah [18], which found that more than 70% of adult diabetic patients scored positive attitude levels toward diabetic foot. This lower level of positive attitude toward diabetic foot needs further qualitative research, through which we can understand the causes of this negative attitude and remove associated barriers, as patient motivation to certain care is the guide for good health. In addition, the current study discovered that married people and those with higher educational levels showed more favorable attitudes regarding diabetic foot, which is consistent with another study that reported the same relationship [31]. Our explanation for this finding is that patients who live with family receive more care and emotional support than single patients. In addition, well-educated people are able to understand their disease well and are keen to prevent its complications; therefore, they can positively deal with their disease.

The study discovered that 77.7% (n = 313) of diabetic patients had poor foot care practices, as fewer than half of them (49.1%, n = 198) regularly inspected their feet. Only 36.7% (n = 148) were interested in detecting changes in foot size, whereas just 18.9% (n = 76) checked the temperature of the water before washing their feet. This is a concerning finding because inappropriate behaviors can contribute to the development of DFU complications. This level of poor foot care practice among diabetic patients matches that observed in another Saudi study [27]. By contrast, another Saudi study in Riyadh found superior diabetic foot care among its sample [29]. This variability in the practice level of foot care in the different Saudi studies may be related to differences in the studies' sample characteristics.

The data from this study identified that diabetic patients with a shorter duration of DM exhibited better practices of foot care, which is consistent with previous research [7]. This may be due to the concerns of newly diagnosed diabetic patients about the complications of DM, which makes them worry about it and adhere to its preventive measures. In addition, patients with DM who have higher educational attainment tend to maintain better care of their feet than those with lower education levels, and they may even postpone the development or recurrence of foot ulcers. These findings are consistent with previous research [3,8,12]. This underscores the role of education in empowering patients to adopt effective foot care practices, while patients with longer DM durations may benefit from continuous reinforcement and support to maintain good practices.

Strengths and limitations

This study gave us an opportunity to highlight the defects in diabetics' knowledge and attitudes about DFUs and their manifestations and complications. Furthermore, it paid attention to the poor foot care among diabetic patients in the Al-Qunfudah district, Saudi Arabia. These findings can direct policymakers to implement more preventive measures among diabetic patients in this geographically isolated area. Despite the advantages of this study, it evaluated foot care practice by asking patients without checking the actual practice, which was considered a limitation in this study. It was better to conduct direct observations of diabetic patients while performing foot care and evaluate the practice score through defined items in a checklist.

## Conclusions

Patients with DM had inadequate foot care practices and inadequate knowledge and negative attitudes concerning DFUs. Around 17% of patients with DM in the Al-Qunfudah district of Saudi Arabia self-reported having a history of DFUs. All diabetic patients need specific training programs to increase their understanding of diabetic foot care and to inspire them to take adequate care of their feet to avoid the occurrence of DFUs; the elderly, female patients, and diabetic patients who are not employed should receive particular attention. Personalized diabetes treatment plans have to be created in order to enhance foot care behaviors, especially for those with DM who are illiterate and have had DM disease for a longer duration. Providing diabetic patients with simple educational materials to help them and their caregivers follow instructions for a DM management plan and procedures for foot care at home and warning signs of diabetic foot. Further qualitative investigation is needed for an accurate understanding of the reasons behind patients' unfavorable opinions of diabetic foot.
